# LEVERAGING TECHNOLOGY TO OPTIMIZE THE ENGAGEMENT OF VIRTUAL PARTICIPANTS IN A NATIONAL CONSENSUS CONFERENCE

**DOI:** 10.1093/geroni/igac059.286

**Published:** 2022-12-20

**Authors:** Francesca Falzarano, Lilla Brody

**Affiliations:** Weill Cornell Medicine, Douglaston, New York, United States; Weill Cornell Medicine, New York, New York, United States

## Abstract

The COVID-19 pandemic has accelerated the uptake and use of technology in hosting virtual and hybrid (combining virtual and in-person) meetings and events. In this presentation, Dr. Falzarano will describe the ways in which the 2021 Conference on Engaging Family and Other Unpaid Caregivers of Persons with Dementia in Healthcare Delivery leveraged videoconferencing systems (e.g., Zoom) and other web-based platforms (e.g., Qualtrics) to enact a hybrid event. Specifically, she will discuss the various components involved in the event’s planning and execution, including the appointment of a virtual liaison and audiovisual technicians to enable seamless integration and participation of both in-person and virtual attendees. She will also discuss how videoconferencing technology was used to facilitate the delivery of virtual panel presentations; strategies for immersing virtual attendees in both large group discussions and small group breakout sessions; and the process by which virtual attendees participated in the priority vote.

